# Caloric Restriction Protects against Lactacystin-Induced Degeneration of Dopamine Neurons Independent of the Ghrelin Receptor

**DOI:** 10.3390/ijms18030558

**Published:** 2017-03-04

**Authors:** Jessica Coppens, Eduard Bentea, Jacqueline A. Bayliss, Thomas Demuyser, Laura Walrave, Giulia Albertini, Joeri Van Liefferinge, Lauren Deneyer, Najat Aourz, Ann Van Eeckhaut, Jeanelle Portelli, Zane B. Andrews, Ann Massie, Dimitri De Bundel, Ilse Smolders

**Affiliations:** 1 Research Group Experimental Pharmacology (EFAR/FASC), Center for Neurosciences (C4N), Vrije Universiteit Brussel (VUB), Laarbeeklaan 103, 1090 Brussel, Belgium; jescoppe@vub.ac.be (J.C.); thomas.demuyser@vub.ac.be (T.D.); laura.walrave@vub.ac.be (L.W.); giulia.albertini@vub.ac.be (G.A.); joeri.van.liefferinge@vub.ac.be (J.V.L.); najat.aourz@vub.ac.be (N.A.); aveeckha@vub.ac.be (A.V.E.); jeanelle.portelli@vub.ac.be (J.P.); 2 Research Group Pharmaceutical Biotechnology and Molecular Biology (MICH), Center for Neurosciences (C4N), Vrije Universiteit Brussel (VUB), Laarbeeklaan 103, 1090 Brussel, Belgium; ebentea@vub.ac.be (E.B.); lauren.deneyer@vub.ac.be (L.D.); ann.massie@vub.ac.be (A.M.); 3 Department of Physiology, School of Biomedical and Psychological Sciences, Monash University, Clayton, Melbourne 3800, Australia; jacqueline.bayliss@monash.edu (J.A.B.); zane.andrews@monash.edu (Z.B.A.)

**Keywords:** Parkinson’s disease, caloric restriction, lactacystin, ghrelin receptor

## Abstract

Parkinson’s disease (PD) is a neurodegenerative disorder, characterized by a loss of dopamine (DA) neurons in the substantia nigra pars compacta (SNc). Caloric restriction (CR) has been shown to exert ghrelin-dependent neuroprotective effects in the 1-methyl-4-phenyl-1,2,3,6-tetrathydropyridine (MPTP)-based animal model for PD. We here investigated whether CR is neuroprotective in the lactacystin (LAC) mouse model for PD, in which proteasome disruption leads to the destruction of the DA neurons of the SNc, and whether this effect is mediated via the ghrelin receptor. Adult male ghrelin receptor wildtype (WT) and knockout (KO) mice were maintained on an ad libitum (AL) diet or on a 30% CR regimen. After 3 weeks, LAC was injected unilaterally into the SNc, and the degree of DA neuron degeneration was evaluated 1 week later. In AL mice, LAC injection significanty reduced the number of DA neurons and striatal DA concentrations. CR protected against DA neuron degeneration following LAC injection. However, no differences were observed between ghrelin receptor WT and KO mice. These results indicate that CR can protect the nigral DA neurons from toxicity related to proteasome disruption; however, the ghrelin receptor is not involved in this effect.

## 1. Introduction

Parkinson’s disease (PD) is a neurodegenerative disorder affecting around 7 to 10 million people worldwide [1] and is characterized by the degeneration of dopamine (DA) neurons in the substantia nigra pars compacta (SNc) that send their axons mainly to the striatum. The resulting decrease in striatal DA is associated with motor symptoms such as rigidity, bradykinesia, postural instability, and tremor [2]. While less than 10% of all cases have a positive family history, the majority are considered sporadic [3]. Therefore, in addition to a genetic predisposition, a variety of other factors are associated with the development of PD. Animal studies showed that obesity may be a risk factor for PD. Diet-induced obesity aggravates 1-methyl-4-phenyl-1,2,3,6-tetrathydropyridine (MPTP)-induced DA degeneration in the SNc in mice [4,5]. In addition Morris et al. showed that a high-fat diet induced more pronounced neurodegeneration in SNc and more DA depletion in the striatum of 6-hydroxydopamine (6-OHDA)-injected mice compared to a normal diet, possibly due to increased oxidative stress [6]. In contrast to obesity, it was shown that caloric restriction (CR) increases lifespan, delays the onset of age-related diseases, and decreases oxidative stress [7,8,9]. A primate study showed that CR prevents age-related brain atrophy in subcortical regions, such as the caudate and putamen, and the left insula [7]. Furthermore, CR ameliorates neurochemical deficits and motor dysfunction in the MPTP model for PD [10], a model that induces mitochondrial dysfunction and both intracellular- and microglia-mediated oxidative stress [11]. Indeed, CR reduced striatal DA loss and improved locomotor activity following MPTP administration in rhesus monkeys [10]. A study in Caenorhabditis elegans further showed a neuroprotective effect of CR in the 6-OHDA model, an effect mediated via silent information regulator (sir)-2.1, a nicotinamide adenine dinucleotide NAD^+^-dependent deacetylase known to extend lifespan [12]. These findings suggest that changes in energy homeostasis can influence neurodegeneration following toxic stimuli. Other studies, however, failed to observe any significant protective effects of CR on the nigrostriatal DA pathway in the MPTP mouse model [13] or the 6-OHDA rat model of PD [14]. In addition, the effect of CR on coexisting molecular pathways in PD remains presently unknown and is of particular interest given the heterogeneous nature of the pathogenesis in the human disorder [15].

During the early stages of CR, acylated ghrelin levels are significantly elevated [16,17]. Ghrelin, a 28-amino acid peptide, is the endogenous ligand of the ghrelin receptor 1a (formerly known as growth hormone secretagogue receptor 1a) [18]. It is mainly produced by the X/A like cells of the stomach, where it is acylated at the third amino acid residue (serine) by ghrelin-*O*-acyltransferase (GOAT). This acylation step has long been considered necessary for its binding to the ghrelin receptor, although some recent studies show that des-acyl ghrelin, which lacks the acyl group, can function via the ghrelin receptor [19,20]. In the brain, in situ hybridization showed that the ghrelin receptor is present in the pituitary gland, the arcuate nucleus, the hippocampus, the median and ventral raphe nuclei, and the midbrain, such as the SNc and the ventral tegmental area (VTA) [21,22]. Ghrelin is known as a regulator of energy balance. In the hypothalamus, ghrelin binds the orexigenic Agouti-related peptide-containing neurons, which consequently leads to an increase in food intake [23]. Surprisingly, genetic ablation of ghrelin or the ghrelin receptor following a normal diet or CR regimen, shows no or modest changes in food intake and body weight, thereby suggesting that compensatory mechanisms may be involved to control feeding [24,25,26,27].

Ghrelin enhances dopaminergic neuron firing in the SNc and increases dopamine release and turnover in the striatum [28,29]. Studies further showed an important role for ghrelin and its receptor in the neuroprotection of DA neuron cell death. Exogenous ghrelin administration could protect SNc DA neurons from MPTP-induced cell degeneration by blocking apoptotic pathways and microglial activation [30,31,32]. This effect seems to be mediated via the ghrelin receptor, given that concurrent administration of ghrelin receptor antagonist d-Lys-3-GHRP-6 abolished the protective effects of ghrelin in this model [30]. In addition, one study demonstrated that ghrelin enhances uncoupling protein 2 (UCP2) activity in the mitochondria of DA neurons, thereby reducing oxidative stress in an MPTP-targeted cell [29]. Further evidence in knockout (KO) studies showed that ghrelin receptor KO mice are more sensitive to MPTP-induced neurotoxicity, while reactivation of the ghrelin receptor specifically in nigral DA neurons reverses this effect [29], further suggesting a protective role for ghrelin receptor action in this model. Recently, an increase in ghrelin levels following CR has been proposed to mediate the neuroprotective effects of this dietary intervention against the loss of nigral DA cells following MPTP administration in mice [33]. This finding is in line with the previously reported protective effects of exogenous ghrelin administration in MPTP models [29,30,32], which were recently attributed to the actions of acylated (rather than des-acyl) ghrelin [34]. In a separate study, ghrelin was found to protect MES23.5 DA-ergic cells against rotenone toxicity [35]. These findings suggest that ghrelin and ghrelin receptor activation are protective in models of PD, an effect that might be mediated by the stimulation of AMP-activated protein kinase (AMPK) activity [33], the enhancement of mitochondrial bioenergetics [29], or the inhibition of microglial activation [32].

PD includes different molecular mechanisms (mitochondrial dysfunction, oxidative stress, deficiency of ubiquitin-proteasome system) that contribute to disease progression. So far, only symptomatic treatment is available for patients. One of the possible reasons for the inability to translate preclinical findings to patient treatment is the poor diversity of animal models used during drug validation [36]. For this reason, it is of great importance to test preclinical findings in animal models for PD with different molecular mechanisms of actions.

The proteasome inhibition model of PD is a relatively new toxin-induced model, based on the intracerebral administration of proteasome inhibitors, such as lactacystin (LAC), to the nigrostriatal pathway [37]. This approach leads to a build-up of cytosolic proteins, including disease-linked proteins such as α-synuclein, triggering DA-ergic dysfunction and nigral DA-ergic cell death [38]. This contrasts with other PD models such as the MPTP model, which causes mitochondrial dysfunction and increases oxidative stress [39].

Apart from the MPTP and rotenone models, which are strongly dependent on mitochondrial inhibition, less is known regarding the putative protective effects of CR against neurotoxins that trigger alternative pathways. Several studies showed that caloric restricted animals rescue 26S proteasome activity in the brains of aged rats and display increased autophagy in cortical neurons through the ghrelin receptor and also in other tissues [40,41,42]. Pan and co-workers showed that a link exists between autophagy, a mechanism that is primarily responsible for the recycling of damaged organelles and aggregated proteins, and proteasome impairment by demonstrating that rapamycin, an autophagy enhancer, significantly attenuated the lactacystin-induced loss of nigral DA neurons. This means that an increase in autophagy activity can enhance the degradation of misfolded proteins and can protect nigral DA neurons from proteasome inhibition-induced cell death [43]. We therefore decided to investigate the potential neuroprotective effect of CR in the lactacystin mouse model. One study in a cellular model of Alzheimer’s disease further showed that ghrelin is also able to increase protein degradation via the ubiquitin-proteasome system [44]. In addition to several studies in the MPTP model suggesting that ghrelin or ghrelin receptor activation might mediate this neuroprotective effect, we also tested the hypothesis that the ghrelin receptor is necessary for the protective effects of CR against the proteasome inhibition-induced loss of nigral DA neurons.

Finally, we studied whether, under physiological conditions, genetic deletion of the ghrelin receptor induces the degeneration of the nigrostriatal tract. Our study showed that CR protects against lactacystin-induced DA-ergic degeneration, independent of the ghrelin receptor.

## 2. Results

### 2.1. Deletion of the Ghrelin Receptor Does Not Induce DA-Ergic Degeneration in the SNc of Adult and Aged Mice

**Figure 1:**
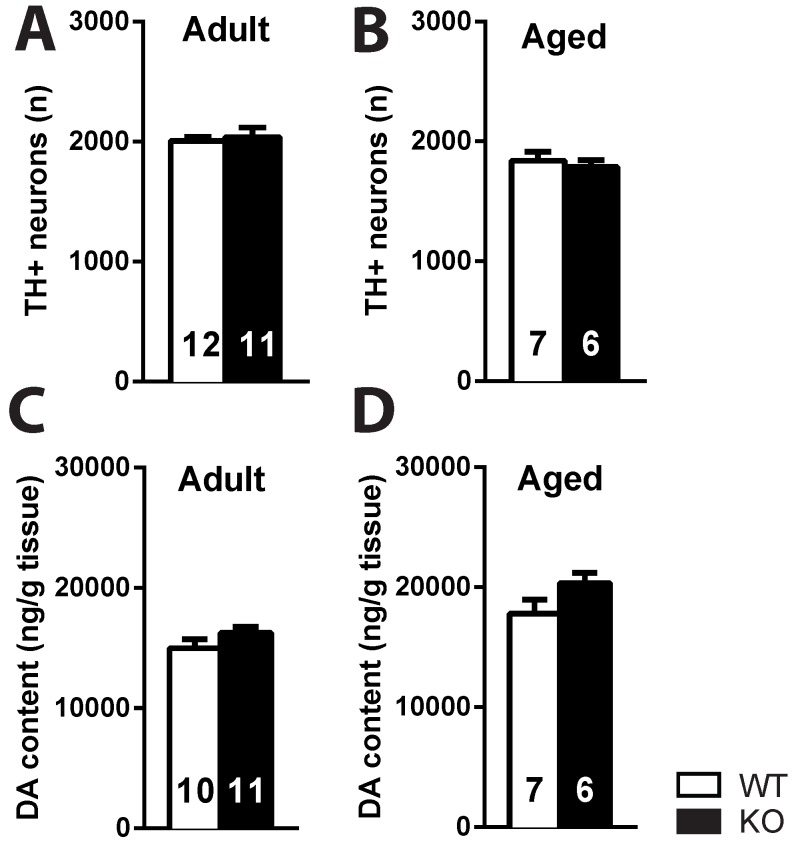
The nigrostriatal pathway of ghrelin receptor knockout (KO) mice is intact. No difference in the number of TH+ profiles in the substantia nigra pars compacta (SNc) (average of left and right SNc) (**A**,**B**) or striatal dopamine (DA) concentration (average of left and right striatum) of adult and aged ghrelin receptor wildtype (WT) and knockout (KO) mice (**C**,**D**). *p* > 0.05 (*t*-test).

No difference was detected in the number of TH+ neurons in the SNc of adult (3–4 months) or aged (18–22 months) ghrelin receptor KO mice and their WT littermates (*p* > 0.05) (Figure 1A,B). Also, striatal DA content did not change between ghrelin receptor WT and KO mice (*p* > 0.05), independent of age (Figure 1C,D).

### 2.2. No Effect of Ghrelin Receptor Ablation on Metabolic Parameters during CR

**Figure 2:**
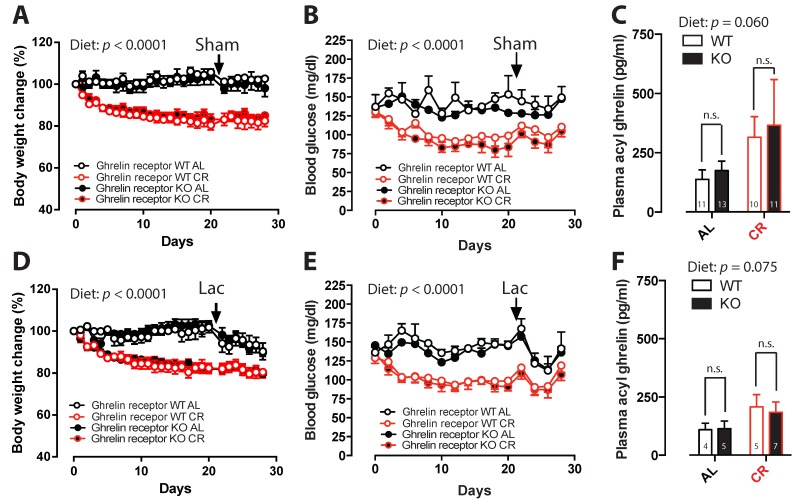
The effect of caloric restriction on (**A**,**D**) body weight (relative to day 0); (**B**,**E**) blood glucose levels; (**C**,**F**) and plasma acyl-ghrelin levels in sham- (**upper** panels) and LAC-injected (**lower** panels) ghrelin receptor WT and KO mice under a caloric restriction (CR) or ad libitum (AL) feeding regimen. The arrow shows the day of intranigral sham or lactacystin (LAC) injection. (Two-way ANOVA followed by a post-hoc Sidak’s multiple comparison test, panel **A**,**D**,**E**: Diet (AL versus CR) *p* < 0.0001, time-effect *p* < 0.0001; panel **B**: Diet (AL versus CR) *p* < 0.001, time-effect *p* < 0.0001; panel **C**: Interaction *p* = 0.913, Diet (AL versus CR) *p* = 0.060, Genotype *p* = 0.554; panel **F**: Interaction *p* = 0.747, Diet (AL versus CR) *p* = 0.075, Genotype *p* = 0.831).

Under CR conditions, saline-injected ghrelin receptor WT and KO mice lost around 16% of their initial body weight (BW) (Figure 2A), and blood glucose (BG) levels diminished by 15%–20% (Figure 2B). LAC-injected WT and KO mice, that underwent 4 weeks of CR, lost ~20% of their initial BW (Figure 2D), and BG levels decreased by 8% and 20%, respectively (Figure 2E). Plasma acyl-ghrelin levels showed a tendency to increase in all CR experimental groups compared to ad libitum (AL) groups (*p* = 0.06 in sham mice, *p* = 0.075 in LAC lesioned mice) (Figure 2C,F). It seems as if the effect of CR on acyl ghrelin levels appears lower in lactacystin-treated animals; however, when analyzing the data together, no statistical difference was found [45]. Overall, the deletion of the ghrelin receptor did not induce changes in BW, BG, or plasma acyl-ghrelin levels during CR or when fed AL.

### 2.3. Caloric Restriction Inhibits Nigral Cell Loss Following LAC Administration

**Figure 3:**
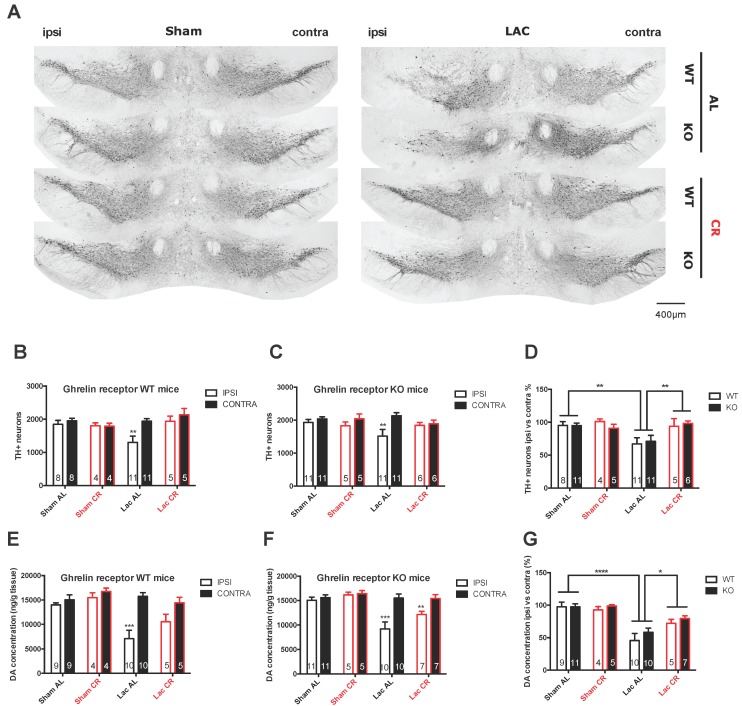
Degeneration of the nigrostriatal pathway in sham- and LAC-injected ghrelin receptor WT and KO mice fed AL or under CR. (**A**) Representative photomicrographs of tyrosine hydroxylase (TH) staining in the SNc of ghrelin receptor WT and KO mice under AL or CR feeding conditions seven days post-lesion (scale bar = 400 µm); (**B**–**D**) The number of TH+ neurons and percentage of TH+ cell loss of the ipsilateral SNc compared to the contralateral SNc seven days after LAC administration in CR and AL-fed WT and KO mice; ** *p* < 0.01 versus contra (**B**,**C**); Two-way ANOVA followed by a post-hoc Sidak’s multiple comparison test) or ** *p* < 0.01 versus sham AL or LAC CR (**D**); Two-way ANOVA followed by a post-hoc Sidak’s multiple comparisons test) (**E**–**G**); DA concentration (ng/g tissue) and percentage of DA loss in the ipsilateral striatum compared to the contralateral striatum seven days after LAC administration in CR and AL-fed ghrelin receptor WT and KO mice. *** *p* < 0.001 versus contra (**E**–**F**); Two-way ANOVA followed by a post-hoc Sidak’s multiple comparison test or * *p* < 0.01, *** *p* < 0.001 versus sham AL or LAC CR (**G**); Two-way ANOVA followed by a post-hoc Sidak’s multiple comparison test.

Intranigral LAC administration significantly decreases the number of TH+ profiles in the ipsilateral SNc compared to the contralateral SNc both in AL-fed ghrelin receptor WT (*p* < 0.01) and in AL-fed ghrelin receptor KO mice (*p* < 0.01) (Figure 3A–C). Furthermore, significant loss of DA-ergic neurons was also observed in the LAC AL group when compared to the sham AL group (*p* < 0.01) (Figure 3D). Interestingly, dopaminergic neurodegeneration was not observed in the LAC CR group compared to the sham AL or CR group (*p* > 0.05), and a significant increase in TH+ neuron survival was visible compared to the LAC AL group (*p* < 0.01) (Figure 3D). Overall, no differences could be detected between ghrelin receptor WT and ghrelin receptor KO mice when investigated for sensitivity to LAC injection, either in the AL or CR groups.

### 2.4. Caloric Restriction Limits Striatal DA Loss in LAC-Injected Mice

At 7 days after LAC injection in the SNc, a decreased DA concentration in the ipsilateral striatum was observed when compared to the contralateral striatum in AL-fed mice (*p* < 0.0001) (Figure 3E–G), and the relative loss (ipsilateral versus contralateral) was significantly higher in LAC-injected mice compared to sham-injected AL-fed mice (*p* < 0.001) (Figure 3G). At 4 weeks after CR, LAC-injected mice showed a higher striatal DA concentration compared to LAC-injected AL-fed mice (*p* < 0.05) (Figure 3G). Again, genetic ablation of the ghrelin receptor did not induce any changes in striatal DA loss following LAC lesion, either in the AL or CR groups.

## 3. Discussion

As previously reported, our present study confirms that intranigral LAC injection in mice in a dose of 3 µg significantly induces nigral DA-ergic cell loss and decreases striatal DA concentration [38]. We show for the first time that CR can significantly inhibit nigral DA neuron loss and partially protect striatal DA content following LAC lesioning. CR was shown before to exert neuroprotective effects in several PD models. In both a primate (MPTP), mouse (MPTP), and a Caenorhabditis elegans (6-OHDA) model for PD, CR had a preventive effect on DA-ergic neurodegeneration [10,33,46]. Together with our study, we further evidence to strengthen the hypothesis that CR can protect nigral DA neurons from neurodegeneration. As CR shows a neuroprotective effect in several PD models (MPTP, LAC, and 6-OHDA), this makes CR an interesting target for further investigation to find a disease-modifying therapy. However, clinical trials studying the effect of CR on neurodegeneration will be difficult to perform because of long-term compliance issues; therefore investigators are currently looking for the mechanisms of action of CR-induced protection of nigral DA neurons so that drugs mimicking the beneficial effects of CR can be developed.

When using intermittent fasting, the neuroprotective effect, as was seen with CR,was not that clear as only one study could prove that CR reduced DA-ergic cell loss after injecting MPTP or 6-OHDA [14,47,48]. Hence, it might be critical to distinguish between an intermittent fasting regimen and daily reduced caloric intake when studying the effects of CR in animal models for PD [49].

Using a CR regimen that was reported to elevate plasma ghrelin levels [33] we found a strong tendency towards increased plasma acylated ghrelin levels following CR. Next, we investigated whether ghrelin receptor KO mice would lose the neuroprotective effect of CR after LAC injection. We did not see any significant differences in metabolic parameters between ghrelin receptor WT and KO mice during CR. Furthermore, the deletion of the ghrelin receptor did not alter DA loss in the LAC model during CR. This finding implicates that, in contrast with the MPTP model, ghrelin receptor activation does not play an important role in the CR-induced protection of LAC-induced cell loss. One possible explanation for the discrepant findings between these two models might be linked with their distinct mechanism of action. Of note, in the study by Andrews and colleagues, ghrelin KO mice lost more nigral DA neurons following MPTP administration compared to control mice, suggesting that reduced ghrelin receptor activation could increase the sensitivity of DA neurons to MPTP-induced degeneration [29]. In contrast, in our study, ghrelin receptor KO mice, when fed AL, did not show a differential loss of nigral DA neurons or striatal DA content following LAC administration, suggesting a differential involvement of the ghrelin receptor in MPTP- and LAC-induced neurotoxicity. Furthermore, it is conceivable that alternative mechanisms are recruited by CR, leading to the observed neuroprotective effects. Indeed, CR works through a multitude of actions to achieve a preventive effect in DA-ergic cell loss. Not only ghrelin, but also other peptides such as leptin and adiponectin levels are changed during CR [50].

CR restriction induces the expression of neurotrophic factors such as glial cell line-derived neurotrophic factor (GDNF) or brain-derived neurotrophic factor (BDNF) that could be involved in protecting the nigrostriatal DA pathway against toxic stimuli [10]. Interestingly, it has been reported that hippocampal BDNF expression is increased during CR to similar extents in both ghrelin KO and WT mice [51]. Further investigation to examine the contribution of neurotrophic factors to the CR-induced neuroprotective effect following LAC lesioning is thus warranted.

Another possible mechanism of action of CR is the sirtuin 1-autophagy pathway. Previous studies showed that CR can induce autophagy through enhancement of AMPK and Sirtuin 1 activity, thereby decreasing protein aggregation and increasing longevity [52,53]. Although Bayliss et al. showed that ghrelin signaling through AMPK mediates the neuroprotective effects of CR in the MPTP mouse model [33], we showed a ghrelin receptor-independent neuroprotective effect of CR in our LAC model. A possible explanation is that sirtuin 1-mediated effects can occur independently of AMPK activity, as shown by Song et al. who demonstrated that metformin protects against MPTP through Sirtuin 1 and autophagy but acts independently of AMPK [54]. Although speculative, the neuroprotective effect of CR in this study could thus be induced via increased sirtuin 1 activity and subsequent autophagy, independent of the ghrelin receptor and AMPK activity, thereby decreasing LAC-induced protein aggregation and neurodegeneration.

An alternative, but not mutually exclusive, hypothesis is that the increase in plasma ghrelin levels observed after CR in our group of mice might have been insufficient to stimulate ghrelin receptor activation in the SNc. This could explain the lack of involvement of the ghrelin receptor following CR, especially as the protective effects of ghrelin in the MPTP model were found to be dose-dependent [30]. Although we employed a similar CR protocol previously reported to lead to a robust increase in plasma acylated ghrelin levels [16,33], the effect was more modest in our study and particularly visible in the sham ghrelin receptor WT group. This variable effect on plasma ghrelin levels might be linked with the differences in the genetic background of the mice used in the present study (mixed 129Sv/Evbrd(LEX1)/C57BL/6 background) compared to the previously cited studies (C57BL/6J background) [33,55].

Another possibility that can explain why we do not see a difference in neuroprotection between ghrelin receptor WT and KO mice is that des-acyl ghrelin induces neuroprotection in the LAC mouse model. A previous study showed that in vitro des-acyl ghrelin inhibits oxygen-glucose deprivation-induced cell death in cultured neurons [56]. In vivo des-acyl ghrelin administration showed a protective effect after ischemic injury [57]. It is however uncertain to which receptor des-acyl ghrelin binds. Some studies show that des-acyl ghrelin is able to interact with the ghrelin receptor [19,20], while other studies suggest that des-acyl ghrelin interacts with another unknown receptor [56,57]. If des-acyl ghrelin would interact with a different receptor from acylated ghrelin, it is possible that increased des-acyl ghrelin is able to induce neuroprotection, even in ghrelin receptor KO mice. However, in the MPTP mouse model it was demonstrated that it was acylated ghrelin, and not des-acyl ghrelin, that protected nigral DA neurons from cell death [34].

Of note, ghrelin has recently been shown to stimulate the function of the proteasome pathway in cellular models of Alzheimer’s disease [44]. Whether or not exogenous ghrelin administration would protect nigral DA neurons against proteasome inhibition-induced cell death remains an important question to address in future studies, especially with the prospect of this strategy to afford neuroprotection in PD [58].

As an important control experiment we checked for possible spontaneous neurodegeneration by ghrelin receptor deletion; nevertheless, we also did not see changes in TH+ neurons in adult or aged ghrelin receptor KO mice versus their WT littermates. Thus it seems that the absence of the ghrelin receptor does not induce nigral degeneration.

Together with our study, it is clear that CR shows a neuroprotective effect in several animal models with different mechanisms of action; hence this implies that CR remains an interesting path to further investigate and thereby unravel which mechanisms are behind this neuroprotective effect. Unfortunately, we were not able to confirm in this model that the CR-induced neuroprotective effect is mediated via the ghrelin receptor, making it not the main factor involved in the CR-induced neuroprotective effect in the LAC mouse model. As the neuroprotective effect of ghrelin in the MPTP mouse model could not be reproduced in the LAC mouse model, it remains dubious whether the ghrelin axis could be a potential target for future treatment in PD.

## 4. Material and Methods

### 4.1. Animals

All experiments were carried out according to the National guidelines on animal experimental research and the procedures were approved by the Ethical Committee for animal experiments of the Vrije Universiteit Brussel (CEP14-213-7, ethical permission date: 14 Feb 2014; CEP 13-275-1, ethical permission date: 01 Mar 2013). Male adult (3–4 months) and aged (18–22 months) ghrelin receptor WT and ghrelin receptor KO mice on a mixed 129Sv/Evbrd(LEX1)/C57BL/6 background were used for this study. The ghrelin receptor KO mice were created by Janssen Pharmaceutica (Beerse, Belgium) in collaboration with Lexicon Genetics, Inc (The Woodlands, Texas, USA), as previously described [59]. Genotypes were confirmed by PCR of tail DNA, using the Terra PCR direct genotyping kit (Westburg, the Netherlands). The following primers were used for ghrelin receptor WT mice: 5′-TGGGGGTGCGAACATTAGC-3′ and 5′-CTGAAGGCATCTTTCACTACG-3′; and for ghrelin receptor KO mice: 5′-ACATATTCTATGTGAGGCACC-3′ and 5′-CTGAAGGCATCTTTCACTACG-3′. All mice were housed under standard conditions (25 °C, 14/10 h light/dark cycle) and were fed ad libitum (AL) before the start of the experiment.

### 4.2. Possible Neurodegeneration by Ghrelin Receptor Ablation

A group of adult ghrelin receptor WT and ghrelin receptor KO littermates and a group of aged ghrelin receptor KO and WT littermates were fed AL and were sacrificed without any further intervention (no surgery) to investigate the effect of the genetic ablation of the ghrelin receptor on the neurodegeneration of the nigrostriatal pathway.

### 4.3. Caloric Restriction

Before assigning the mice to their feeding regimen, the adult mice were single housed and their food intake was measured for 5 days. Starting 21 days prior to LAC or sham injection, mice undergoing CR received 70% of normal food consumption, based on their individually-calculated daily food intake. Tap water was available AL for both the CR- and AL-fed groups. Mice were weighed and fed daily around 2 h before the onset of the dark cycle. Blood glucose (BG) was measured every 2 days prior to feeding. Mice remained on the feeding regimen until the termination of the experiment; 28 days in total. On the 28th day, body weight (BW) and BG were measured immediately before sacrifice.

### 4.4. Nigral LAC Lesioning

On the 21st day of the feeding regimen, mice underwent surgery as previously described [38]. Briefly, mice were anesthetized with a mixture of ketamine (100 mg/kg i.p.; Ketamine 1000 Ceva, Ceva Sante Animale, Brussels, Belgium) and xylazine (10 mg/kg i.p.; Rompun 2%, Bayer Naamloze Vennootschap, Brussels, Belgium) and positioned in a Kopf Model 963 Ultra Precise Small Animal Stereotaxic Frame with a mouse adaptor (David Kopf Instruments, Tujunga, CA, USA). The skull was exposed, and a small hole was made through the skull above the left SNc. A volume of 1.5 μL LAC 2 μg/μL was injected into the left SNc at the following coordinates; AP −3.0 mm, LM −1.0 mm, and DV −4.5 mm from bregma, according to the atlas of Paxinos and Franklin [60]. LAC solutions were prepared by dissolving 50 μg LAC (Cayman Chemical, Ann Arbor, MI, USA) in 25 μL NaCl 0.9%. In order to minimize lesion variability due to degradation of the toxin ex vivo, fresh LAC solutions were prepared for every four mice and immediately stored on ice. Control sham-operated mice received the same volume of vehicle (NaCl 0.9%) at the same coordinates. To minimize unspecific tissue damage, microinjections were performed using a 10 μL Model 1701 RN Neuros Syringe (Hamilton Company, Reno, NV, USA) at a flow rate of 0.5 μL/min. After injection, the syringe was left in place for an additional 5 min and then slowly removed. At the end of the surgery, the skin was sutured and mice received 4 mg/kg ketoprofen s.c. (Ketofen, Merial, Brussels, Belgium) for post-operative analgesia. On the day of surgery, CR-restricted animals received 50% of their daily food pellet at 7 am (before surgery). The other 50% was given around 2 h before the onset of the dark cycle. The AL animals received fresh food pellets before and after surgery.

### 4.5. ELISA for Determination of Acetylated Ghrelin

One week after surgery, all mice were sacrificed by cervical dislocation following sedation and trunk blood was collected in ethylenediaminetetraacetic acidEDTA tubes pretreated with Pefabloc SC (final concentration 1 mg/mL, Sigma-Aldrich, Saint Louis, MO, USA). The blood was centrifuged and the collected plasma (supernatant) was treated with 0.05 N HCl. Plasma samples were used for the determination of acylated ghrelin using an ELISA kit according to the manufacturer’s instructions (Millipore, Temecula, CA, USA).

### 4.6. Liquid Chromatographic Determination of Total DA Content in the Striatum

After cervical dislocation, the brains were rapidly removed and the striata were isolated on an ice-cold petri-dish. Next, both the ipsi- and contralateral striatum were weighed and homogenized separately (30 s) in 400 µL antioxidant solution containing 3,4-dihydroxybenzylamine (1 µg/mL) as an internal standard. The homogenates were centrifuged for 20 min at 10,000× *g* at 4 °C and the supernatants were collected and 1:5 diluted in 0.5 M acetic acid. For the determination of total DA content in the ipsi- or contralateral striatum, 20 µL of the mixture was analyzed using narrow-bore (Alltima C18 column, 5 µm, 150 mm × 2.1 mm, Alltech Grace, Lokeren, Belgium) liquid chromatography with electrochemical detection, as previously described [61]. Results are expressed as ng DA/g wet tissue.

### 4.7. Immunohistochemistry for Detection of Tyrosine Hydroxylase (TH)-Containing Neurons

After cervical dislocation, the caudal part of the brain was removed and post-fixed for three days in freshly prepared 4% paraformaldehyde. Free-floating sections of 40 µm were sliced with a vibratome and were stored at 4 °C in 10 mM PBS + 1.5 mM NaN_3_. Nigral sections were sampled throughout the entire rostrocaudal extent of the SNc (2.92 mm until 3.64 mm posterior to bregma) to investigate the presence of TH-expressing neurons in the ipsi- and contralateral SNc. During the staining, all the rinsing steps and all the dilutions were performed in Tris-saline. On the first day of the staining, 3% H_2_O_2_ was administered followed by a blocking step with normal goat serum (1:5, Millipore) and overnight incubation with rabbit anti-TH antibody (AB152; 1/2000; Millipore). On the second day, sections were processed by the avidin-biotin method, using the Vectastain ABC kit (Vector laboratories, Burlingame, CA, USA), and the glucose oxidase-diaminobenzidine-nickel method was used for visualization. All sections were air-dried, cleared in xylene, and coverslipped with Dystirine Plasticer Xylene (DPX). Photomicrographs were made of the stained sections and cell counts of the SNc were performed using ImageJ software (version 1.45s, USA National Institutes of Health, Bethesda, MD, USA). The number of TH+ neurons was counted in six serial sections throughout the entire rostrocaudal extent of the SNc (2.92 mm until 3.64 mm posterior to bregma) (staining of 1 section per 3 sections) by a scientist blinded to the genotype and the feeding regimen. This total number was then multiplied by three.

### 4.8. Statistical Analysis

Statistical analysis was performed using GraphPad Prism 6.0 software. Data are expressed as mean ± SEM. We used a *t*-test to compare the nigral TH+ profiles and striatal DA content in adult and aged ghrelin receptor WT and KO mice. For all other comparisons, a two-way ANOVA followed by Sidak’s multiple comparisons test was used. The α-value was set at 0.05.

## 5. Conclusions

According to our findings, CR has a neuroprotective effect in the LAC mouse model for PD. Our finding is in line with the previous reports that showed a neuroprotective effect of CR in the MPTP and 6-OHDA models [10,12,33]. However, we found no evidence that the CR-induced attenuation of nigral DA neuron loss within the LAC model is dependent on ghrelin receptor-mediated actions.
